# Recent Advances in Elongational Flow Dominated Polymer Processing Technologies

**DOI:** 10.3390/polym13111792

**Published:** 2021-05-29

**Authors:** Zhongke Yuan, Xiaochuan Chen, Dingshan Yu

**Affiliations:** Key Laboratory for Polymeric Composite and Functional Materials of Ministry of Education and Key Laboratory of High-Performance Polymer-based Composites of Guangdong Province, School of Chemistry, Sun Yat-Sen University, Guangzhou 510275, China; yuanzhk3@mail.sysu.edu.cn (Z.Y.); chenxch29@mail2.sysu.edu.cn (X.C.)

**Keywords:** polymer processing, elongational flow, vane extruder, eccentric rotor extruder, numerical simulation

## Abstract

The continuous development of plasticizing conveying methods and devices has been carried out to meet the needs of the polymer processing industry. As compared to the conventional shear-flow-dominated plasticizing and conveying techniques, a new method for processing polymers based on elongational flow was proposed. This new method and the related devices such as vane extruders, eccentric rotor extruders and so on, exhibited multiple advantages including shorter processing time, higher mixing effectiveness, improved product performance and better adaptability to various material systems. The development of new techniques in the field of polymer material processing has opened up a broad space for the development of new plastic products, improved product performance and reduced processing costs. In this review, recent advances concerning the processing techniques based on elongational flow are summarized, and the broad applications in polymer processing as well as some future opportunities and challenges in this vibrant area are elucidated in detail.

## 1. Introduction

During the past few decades, the advancements in plasticizing and conveying methods and the related devices for polymers and polymer composites have been widely demonstrated to provide substantial property enhancements [1,2,3,4,5]. To date, the most commonly applied polymer plasticizing and conveying systems are based on screws, such as single-screw extruders (SSE), twin-screw extruders (TSE), and so on.

In general, all of these processes involve the following functional zones: (a) a solid conveying section, (b) a melting and melt conveying section, and (c) a metering section [1,2]. In the solids conveying zone of the extruder, the solid feeds are compacted within the screw channel by the rotation of the screw to form a solid plug. The conveying mechanism is based on shear flow where the solid materials are transported by frictional drag, which is dependent on the intrinsic material properties (e.g., heat conductivity) and on the friction between the materials and the screw/barrel [2,6]. Thus, a long thermo-mechanical history is needed for solid conveying completion, which entails long processing time and high energy consumption. 

Shear flows have been proven to be less energy-efficient towards both dispersive and distributive mixing when compared to elongational flows [6,7,8,9]. Much effort has been devoted to developing plasticizing and conveying devices that impart elongation-dominated flows, such as the introduction of extensional mixing elements (EME) into the conventional single-screw [10,11] and twin-screw extruders [12,13]. The novel non-screw devices known as vane extruders (VE) are comprised of several vane plasticizing and conveying units [14], and the later-developed eccentric rotor extruders (ERE) are dominated by a continuous elongational flow field [15,16].

In this review, we provide a brief review covering the latest advancements in plasticizing and conveying methods and the related devices based on elongation-dominated flows as well as their applications in polymer processing including single polymer/polymer blends, polymer–inorganic composites, and fiber-reinforced polymer composites. Furthermore, we also highlight some typical examples of practical applications in processing special polymer composites. Lastly, we elucidate some challenges and potential solutions in this vibrant field. We expect that this review and upcoming efforts in this field will provide useful guidelines for developing highly efficient processing of polymer materials based on elongational flows.

## 2. Plasticizing and Conveying Devices, Mechanism, and Simulations

**Figure 1:**
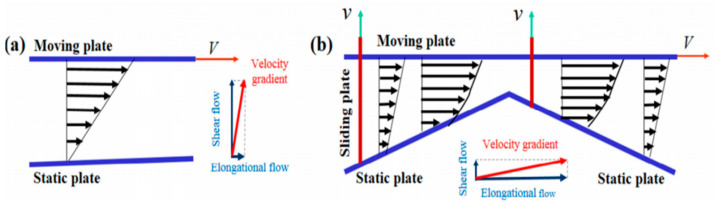
Schematic diagrams of (**a**) shear flow field and (**b**) elongational flow field. Reproduced under the CC BY-NC-ND license from [17].

The plasticizing and conveying process of polymers and their composites, can generally be divided into shear-flow-dominated flow fields and elongational-flow-dominated flow fields. In order to further elaborate the differences between shear-flow-dominated flow field and elongational-flow-dominated flow field, simplified two-plate and three-plate models are presented in the schematic diagrams shown in Figure 1.

Figure 1a exhibits a simplified two-plate model for conventional screw-based plasticizing and conveying devices dominated by a shear flow field, in which the screw and the barrel are simplified as two parallel plates (a moving plate for screws and a static plate for the barrel) and the relative movement of the two plates was simplified as the flow field pattern. Enforced by the drag effect of the moving plate, the materials mainly underwent the process of shear deformation. The major component of the velocity gradient, which occupies the dominant position, is the shear flow. Figure 1b shows the simplified 3-plate model for the elongational flow dominated process. In the plasticizing and conveying process, the volume of materials undergoes repeated (or periodic) change owing to the synchronized movement of the moving plate and the sliding plate, endowing a dominant elongational flow. Although the specific structure of the devices differs, researchers have successfully incorporated the elongational flow elements by setting single or multiple convergence-divergence channels in the systems [6,10,11,12,13,14,15,16]. 

This section will present recent simulations and theoretical advancements regarding various means to incorporate elongational flow in the plasticizing and conveying process.

### 2.1. The Extentional Mixing Element in Conventional Screw Extruders

**Figure 2:**
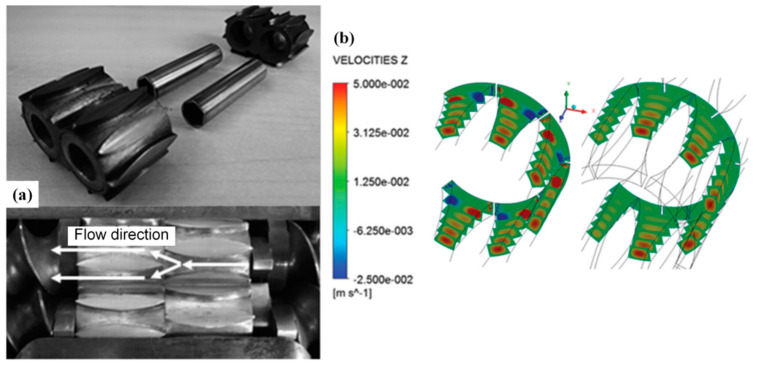
(**a**) Structure of the extensional mixing element (EME) and flow direction in two EMEs in series. (**b**) Velocity profiles for two EME in series. Reproduced with permission from [18]. Copyright 2015, John Wiley and Sons.

As for the introduction of the extensional mixing element (EME) into the conventional SSE and TSE systems, Carson et al. [18] designed the flow channels of the EME in a conventional TSE system, as shown in Figure 2a, to incorporate a specific extension flow on a material through the concentric converging-diverging channels. Simulations of the EME using ANSYS-Polyflow demonstrated that the outlet flow of each diverging channel from the EME unit possessed a linear increase in velocity with much stronger stress components in the flow direction than the shear components (Figure 2b), showing the desired elongational characteristics. Pandey et al. [6] extended the EME concept to SSE systems to fully exploit the good conveying property and enhance the poor mixing capability of SSE. A similar flow domain is established upon finite-element (FE) modeling optimization where biaxial EME showed higher elongation ratio, which was also experimentally validated in various polymer composite systems.

### 2.2. The Vane Extruders

**Figure 3:**
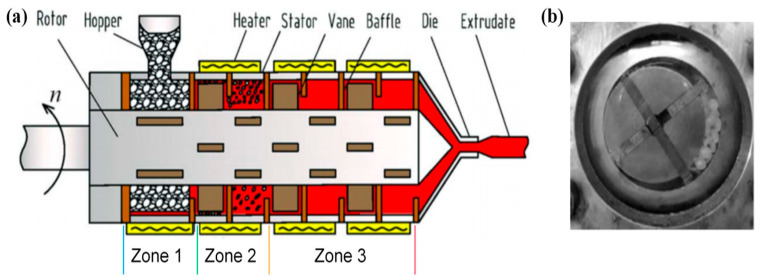
(**a**) Structure of a vane extruder (VE) with different working sections. (**b**) Photograph of the solid compaction zone. Reproduced with permission from [21]. Copyright 2014, John Wiley and Sons.

The basic structures of VEs, which were invented by Qu [19], as shown in Figure 3, include several plasticizing and conveying zones comprised of a cylinder-shaped hollow barrel as the stator, a columned eccentric rotor, and multiple vanes installed on the rotor. When the rotor starts rotating, the space between the vanes and the stator is restricted, subsequently the periodical volume increase and decrease forces the materials through a compaction-mill-discharge process with a short thermo-mechanical history.

Qu and co-workers [20] established a mathematical model regarding the power consumption and pressure building in the solid compaction zone (zone 1 in Figure 3) based on the stress distribution. They found that with increased eccentricity of the vane, the pressure of the compressed solid increased, resulting in an increased total power consumption. According to their study, the major power dissipation is heat transfer and its solid compressing and heating efficiency is better than that of a conventional screw extruder with the same setting parameters. The solid conveying in the solid compaction zone (zone 1) [21] and solid conveying zone (zone 2) [14], as shown in Figure 3, were also theoretically studied and experimentally validated. It was found that the transferred power, despite the general power dissipation in the system (dynamic friction, pressure increase etc.), increases exponentially via the positive conveying, while a steady increase is observed in the static friction dragging. 

Apart from pressure establishment and solid conveying, researchers also studied other characteristics of the VE using modeling and simulations. Xie et al. [22] investigated the energy consumption and mixing characteristics in the melt conveying zone (zone 3 in Figure 3) using computational fluid dynamics, by varying the vane arrangements including four uniformly/non-uniformly distributed vanes, and six uniformly distributed vanes, and tuning the eccentricity of the rotor. It was found that the mixing performance of the VE is proportional to the degree of eccentricity and best performance was exhibited with the four uniformly distributed vanes setting. 

Huang et al. [23] derived the velocity distribution and the mixing characteristics of the solid conveying zone (zone 2 in Figure 3) and examined the velocity profiles of the materials between each vane. It was revealed that the increasing power-law index led to a decreased dimensionless velocity profile which resulted in more efficient material dispersion and uniformity compared to traditional shear-dominated extrusion.

### 2.3. The Eccentric Rotor Extruders

**Figure 4:**
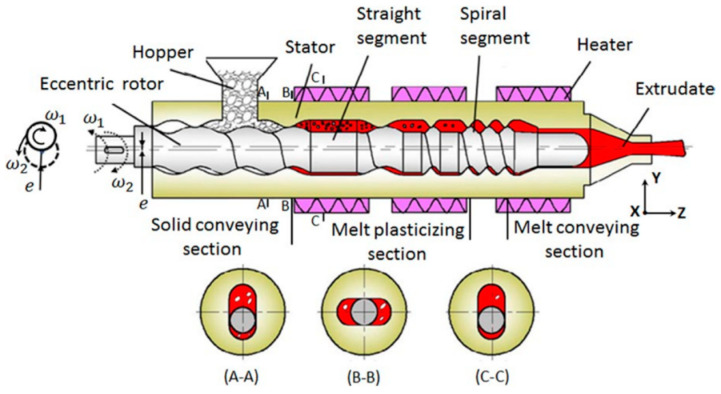
Structure of the eccentric rotor extruder (ERE) with different rotating states. Reproduced with permission from [25]. Copyright 2018, John Wiley and Sons.

Qu and co-workers [24] also developed an elongational-flow-dominated method and devices based on volume pulsed deformation, namely the eccentric rotor extruder (ERE), the basic structure of which is schematically illustrated in Figure 4. The rotation of the spiral-shaped eccentric rotor forced the materials in the space between rotor and stator through multiple converging-diverging zones periodically along both axial and radial directions, hence endowing a plasticizing and conveying process with volume pulsed deformation.

Wen et al. [25] analyzed the flow field in the melt conveying section based on fluid dynamics simulations. Varying technological parameters in the melt conveying section such as rotor speed, radius, and eccentricity in the numerical simulation model, the energy consumption and output efficiency of the ERE were studied. Verified by the demonstration experiment, the simulation analysis revealed that the power of the rotor varied in the same trend with the rotation angle and the production efficiency based on the output efficiency and power consumption is more closely related to the eccentricity of the rotor rather than the rotating speed.

Fan et al. [26] applied particle tracking in the numerical simulation of dispersive and distributive mixing characteristics for the ERE and established a visualization of the mixing process. In the aspect of parameter optimization, a rotation speed of 45 r/min enabled the best mixing performance while increases in the radius and eccentricity of the ERE could also result in improved mixing performance.

While Ultra-high molecular weight polyethylene (UHMWPE) has the same basic molecular structure as that of ordinary polyethylene, its ultra-long molecular chain and exceptionally high entanglement density drastically enhance the interaction between molecules, resulting in its unique properties such as high wear resistance, low friction coefficient, high toughness, and biological inertia. Our group studied UHMWPE and its composites under the elongational flow induced by the ERE, using FE simulations [27], dissipative particle dynamics (DPD) simulations [28,29,30], and experimental validations [31].

**Figure 5:**
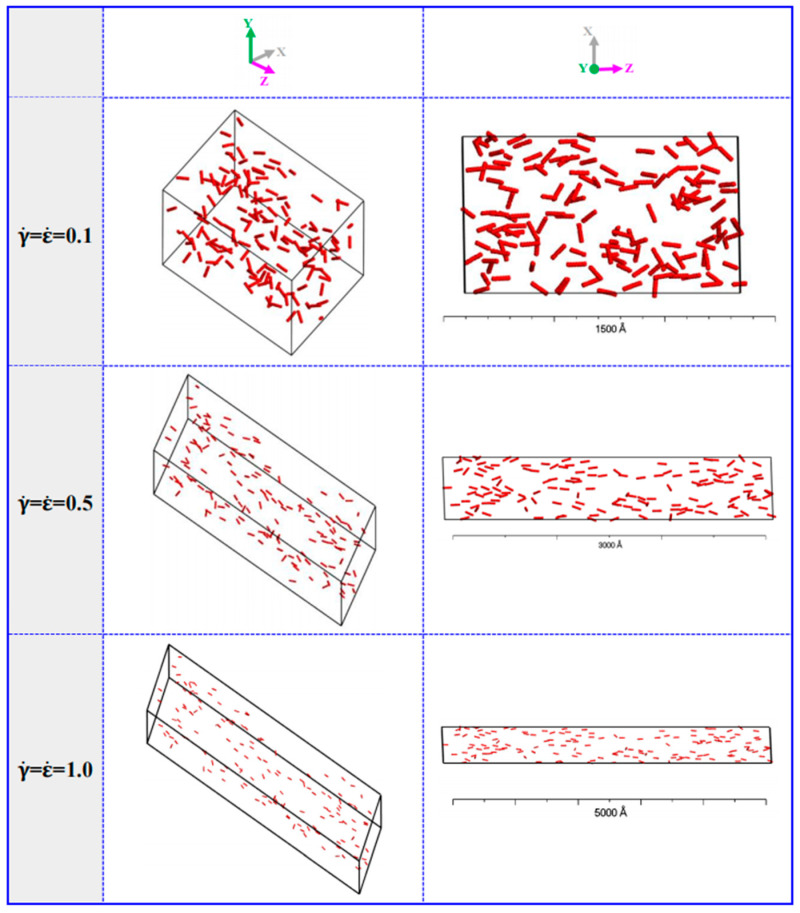
The structure of carbon nanotubes/ultra-high molecular weight polyethylene (CNTs/UHMWPE) composites at 10% CNTs under different extensional–shear coupled rates. The red beads represent CNTs. Reproduced under the Creative Common CC BY license from [28].

We applied the FE simulations in a carbon nanotubes (CNTs)/UHMWPE composite to investigate the effect of various elongational–shear coupled flow field on the deformation and stress response [27]. It was found that both pure elongational flow and shear flow would significantly increase the risk of structural damage. And by tuning the elongational/shear loading ratio (force loading only), a maximum Mises stress in the axial direction and the maximal shear stress were found at 0.45 m and 0.61 m respectively within the pressure ranges of 24–10 MPa and 0.9–0.3 MPa, below the yield strength of the CNT/UHMWPE composite (~29.8 MPa). We further put the microscale structure and interfacial performances of the CNT/UHMWPE system into consideration and employed the DPD simulations to investigate the dispersion and orientation evolution of the CNTs in UHMWPE matrix under various elongational–shear coupled rates [28]. We found that increased elongational/shear loading ratio would result in improved orientation of the CNTs and a more ordered morphology of the composite (as shown in Figure 5). Moreover, this improvement is less dependent on the concentration of CNTs as the mean square displacement of the CNTs in the matrix exhibited slight changes with increased concentrations of CNTs from 3 to 10%.

We also adopted the Souza-Martins method in DPD simulations in search for the predicted flow behaviors of UHMWPE/polyamide 6 (UHMWPE/PA6) blends [29]. By varying the parameters such as mass fractions of UHMWPE/PA6 and elongational/shear coupled rate, it was found that the distribution features of the composite had a minor dependence on the flow variations and mass fractions of the component, yet the orientation behaviors were shifted from micelle-like structure to chain-like structures. The DPD simulations performed in the UHMWPE/polypropylene (UHMWPE/PP) composite [30] showed that the chain conformations differed according to different flow types. Random coils of the polymer chains became collapsed configurations and perpendicular to the flow direction under shear-dominated flows. But by imparting the elongation-dominated coupled flow, the polymer chains appear to be parallel to the flow direction and formed a uniform PP layer.

These findings in theoretical simulations could be of general importance for better in-depth understanding of the mechanism of system evolution and correlations between setting parameters and the properties of the products, and more importantly, can be utilized for device-design optimizations and processing-parameter configurations.

## 3. Applications of Elongational Flow Dominated Plasticizing and Convey Devices

As mentioned in the above section, the elongation-dominated flow has been proved effective to improve the production efficiency of the plasticizing and conveying process and to enhance the products properties of polymers and their composites. Concurrently, much effort has been devoted into the applications of the elongational flow dominated plasticizing and conveying in plural polymer-based systems. The following section describes studies of single polymer/polymer composites, polymer-inorganic composites, and fiber-reinforced polymer composites processed by elongational flow dominated devices. Applications of such devices in processing UHMWPE and its composites is highlighted and discussed.

### 3.1. Single Polymer/Polymer Composites

Free volume between the polymer chains allows a limited mobility of polymer chains under shear flow field or extensional deformation force. Significant improvement in the orientations and crystalline properties of the polymers and the dispersive/distributive uniformity of the polymer blends after the plasticizing and conveying processes, would result in better overall performances of the products.

**Figure 6:**
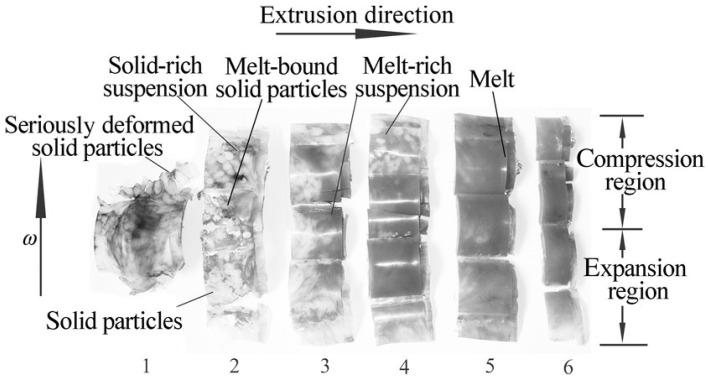
Photographs of high-density polyethylene (HDPE) removed from six VE units. Reproduced with permission for [32]. Copyright 2013, John Wiley and Sons.

Qu et al. [32] studied the melting behavior of high-density polyethylene (HDPE) under controlled variations of eccentricity and rotating speed. By studying the carcass of HDPE removed from different zones in the VE, it was observed that the HDPE pellets underwent different melting mechanisms in different zones of the VE, as demonstrated in Figure 6. In the first few vane units, the HDPE feed was compacted and conveyed under the positive deformation force instead of the friction-induced dragging force in the screw extruders and heated via friction energy dissipation and plastic energy dissipation. With ongoing conveying process, plastic energy dissipation and viscous energy dissipation took place as heat sources, resulting in a deformation mixing-melting with significantly improved processing efficiency. The total length of 6 vane units to reach melt-completion could be as short as 140 mm. Yin et al. [33] also used HDPE to study the impact of the rotation speed and die pressure on the output of VEs, as compared to that of a conventional SSEs. The output of the VE exhibited a linear positive correlation to the rotation speed and independence from the die pressure.

Liu et al. [34] used low density polyethylene (LDPE) to disclose the melting mechanism in elongational flow induced by vane extruders. The melting of LDPE with varied operation parameters (temperature of the heat unit and rotation speed) was examined dynamically by cooling and disassembling the VE units. Unlike the screw-based extruders, after the melt film is formed between the compacted LDPE pellets and the stator in VE, the melts were removed constantly and migrated through voids of LDPE pellets, thus accelerating the heat transfer and dramatically shortening the melting process.

Recently, Wu et al. [35] investigated the melting and crystalline behaviors of recycled poly(ethylene terephthalate) (PET) and comparative analysis was made between ERE and conventional TSE. The molar mass of TSE-processed PET samples decreased significantly more than that of ERE-processed samples, due to longer residence time and much stronger shear component in the flow field, and the laminar thickness of the primary crystallization for ERE-processed PET samples is higher than that for virgin PET samples. As a result, the tensile strength of ERE-processed samples (63.8–66.8 MPa) was slightly improved compared to virgin PET (59.3 MPa), and almost twice that of TSE-processed PET (25.8–31.2 MPa).

The research mentioned above demonstrated the superiority of the elongational flow dominated plasticizing and convey devices over conventional screw-based extruders, in both product performance enhancement and processing efficiency for single polymer.

Polymer blends, the mixtures of different polymers with various properties, is a very effective way to tailor the properties and performances of the polymeric materials. Apart from the intrinsic properties of the selected polymer pairs, the performance of the polymer blends is also determined by the final microstructures [36,37]. However, most of the polymer pairs were immiscible and would lead to unsatisfactory phase transformation such as co-continuous structure. It is well known that the processing methods and the related devices have significant influence on the phase transformation of the processed materials and play important roles in the manipulation the microstructures of final products [35,38,39]. Wu et al. [40] extruded immiscible linear low-density polyethylene (LLDPE)/polystyrene (PS) blends using the VE. By analyzing the phase transformation in the recovery mode, a 325.861% genuine elongational ratio was found, and the morphology development during recovery further indicated that the imposition of the elongational flow field successfully improved the distribution uniformity of the dispersive phase.

He et al. [41] prepared the LLDPE/PET blends using both TSE and ERE and compared the mechanical properties and morphology of different samples. It was revealed that the application of the continuous elongational flow results in better distribution of PET in LLDPE matrix with a certain compatibilizer, exhibiting 226% and 394% improvements in impact strength and elongation at break, respectively. More recently, they successfully improved the properties of metallocene polyethylene (m-PE)/PET blends using ERE [42]. Improvements in both particle/particle size distribution of PET in m-PE matrix were found as compared with the shear flow dominated process in TSE. This research should enlighten new ways of PET modification and applications for recycled PET.

Poly-L-lactic acid (PLA) as a biodegradable and biocompatible polymer, has attracted intensive attentions of researchers. Simple and effective methods to improve the crystallization of the PLA matrix for further mechanical-strength enhancements were proposed [43,44,45,46].

**Figure 7:**
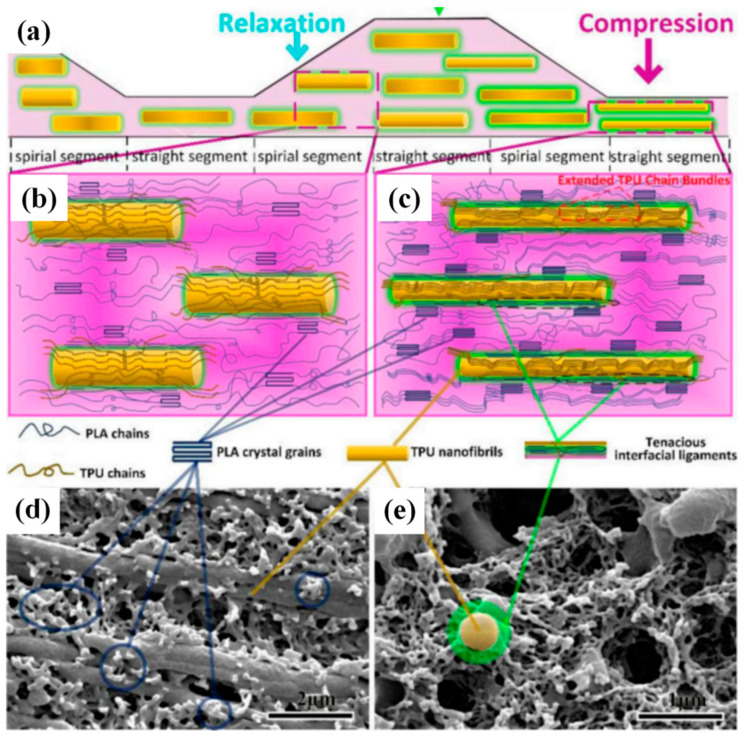
(**a**) Schematic of the formation process of thermoplastic poly(ether)urethane (TPU) nanofibers in ERE. The formation of poly-l-lactic acid (PLA) crystal grains in (**b**) relaxation and (**c**) compression channel. The SEM images of (**d**) etched and (**e**) ultrasonic cleaned fractures. Reproduced with permission from [44]. Copyright 2019, Elsevier.

Elastomeric ethylene-butyl acrylate-glycidyl methacrylate terpolymer (EBA-GMA) was blended with PLA under elongational flow by Fang et al. [43]. The epoxy groups in EBA-GMA are reactive with the end-OH in PLA during the melt blending process and the cross-linking PLA/EBA–GMA interface thus formed contributed to the impact strength enhancement. Meanwhile, the elongational flow field induced the formation of more stable crystal of PLA. Owing to the synergy of the aforementioned mechanisms, the impact strength of the PLA/EBA–GMA blends showed a drastic improvement from ~2.58 kJ m^−2^ to ~55 kJ m^−2^. He et al. studied the reinforcement of PLA from ERE-processed in-situ formed oriented thermoplastic poly(ether)urethane (TPU) nanofibers [44]. As demonstrated in Figure 7, the extended TPU chain bundles in the converging channel worked as nucleating sites, enabling significant improvement in the crystallization of the PLA and the PLA–TPU interfacial adhesion. With an optimized TPU content of 25 wt%, an impact strength of 73.5 kJ m^−2^ was achieved. Further study demonstrated that the interfacial relaxation through the plastic deformation of PLA/TPU matrix endowed a better absorption and dissipation of fracture energy after an annealing process [45]. Thus, reaching a balanced toughness of 90.3 kJ m^−2^ and a stiffness of ~2.15 GPa. Zhang et al. [46] prepared the composite of polycaprolactone (PCL) and PLA using ERE. Under the elongational flow, PLA/PCL composite showed explicit in-situ fiberization and improved compatibility of both components. Optimized composites with a PLA content of 80% reached the highest elongation at break of 476.7%, while the thermal stability was increased by 5.4 °C compared to the neat PLA. 

The improved compatibility between PLA and in-situ fiberized components due to both the increased contacted area [47,48] and the on-fiber nucleating effect [44,45]. And the synergetic enhancement induced by the elongational flow dominated plasticizing and conveying process might be further expanded in other polymer composites. 

**Figure 8:**
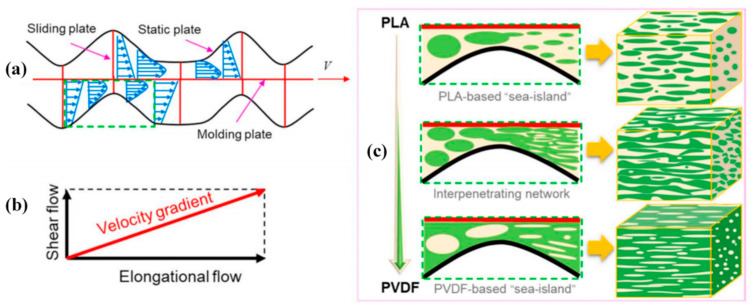
The schematic illustration of (**a**) simplified flow field in twin-eccentric rotor extruder (TERE), (**b**)velocity gradient, and (**c**) morphology evolution of PLA/PVDF composites. Reproduced with permission from [50]. Copyright 2021, Elsevier.

Most recently, on the basis of ERE, the novel twin-eccentric rotor extruder (TERE) was developed [49], and Zhang et al. [50] used TERE to study the mechanism of orientation and dispersion evolution in polyvinylidene fluoride (PVDF) and PLA blends. The melt formed certain “sea-island” regions and an interpenetrating network under repeated breakup and coalescence as demonstrated in Figure 8, and the effective dielectric properties of PLA/PVDF were achieved by varying PVDF ratio.

As mentioned, UHMWPE exhibited extremely high melt viscosity and near-zero melt mobility in the plasticizing and conveying process, putting the conventional shear-flow-dominated processing techniques in a dilemma. Researchers [31,51,52] explored the applications of elongational-flow-dominated plasticizing and conveying devices in UHMWPE processing and significant advancements were made.

**Figure 9:**
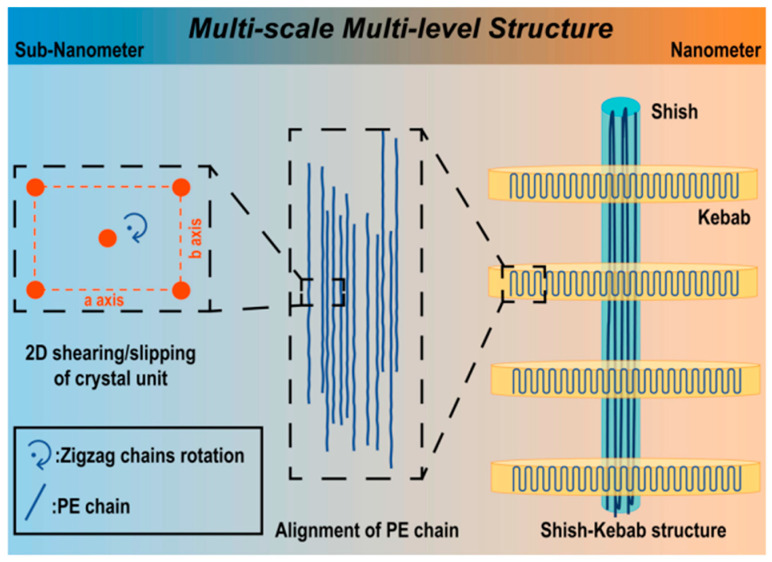
Schematic illustration of PE/UHMWPE prepared under elongational flow. Reproduced with permission from [52]. Copyright 2020, American Chemical Society.

Zhang et al. [51] for the first time successfully plasticized and extruded the UHMWPE without additives using the ERE. They found that the elongational volume deformation enforced a continuous change of the volume of UHMWPE, leading to a better exposure of the unmelted “core” to the heat source, and dramatically enhancing the heating and mass transfer efficiency. Our group [31] performed further comparative analysis of UHMWPE processed under elongational flow and shear flow. We found that the rotation of UHMWPE nascent powder was restrained under the intensive extensional volume deformation, which led to the increased entanglement in the final defect-free products. The shorter residence time of UHMWPE in the extruder also enabled a well preserved molecular weight of the UHWMPE extrudant compared to the UHMWPE nascent powders. Recently, Huang et al. [52] prepared the PE/UHMWPE composite using ERE with a view towards high-voltage applications. The study showed that the PE/UHMWPE formed a shish-kebab multilevel structure, as schematically illustrated in Figure 9, and more intensive elongational flow would lead to more aligned polymer chains in this structure. Compared with conventional cross-linked PE, the PE/UHMWPE showed a higher dielectric constant and a higher dielectric breakdown voltage. 

Based on the studies mentioned above, it is evident that elongational flow dominated processing techniques could not only improve the production efficiency but also bring enhanced properties and expanded applications of polymer/polymer composites.

### 3.2. Polymer-Inorganic Composites

In recent decades, polymer-inorganic nanocomposite materials have been widely reported with substantial enhancements in both chemical and mechanical properties. However, aggregation of nanoparticles has been one of the major issues that hinders exploiting the full potential of polymer-inorganic nanocomposites. To date, researchers have applied elongational volume deformation enforced by the elongational flow dominated plasticizing and conveying devices in plural polymer-inorganic composites with various nanofillers, such as montmorillonite (MMT) [53,54], organically modified montmorillonite (OMMT) [16,55,56,57], titanium dioxide (TiO_2_) [54,58], calcium salt [59,60], and carbon nanotubes (CNT) [54,61], realizing high dispersion effectiveness or even in-situ exfoliation of reaggregated nanofillers.

**Figure 10:**
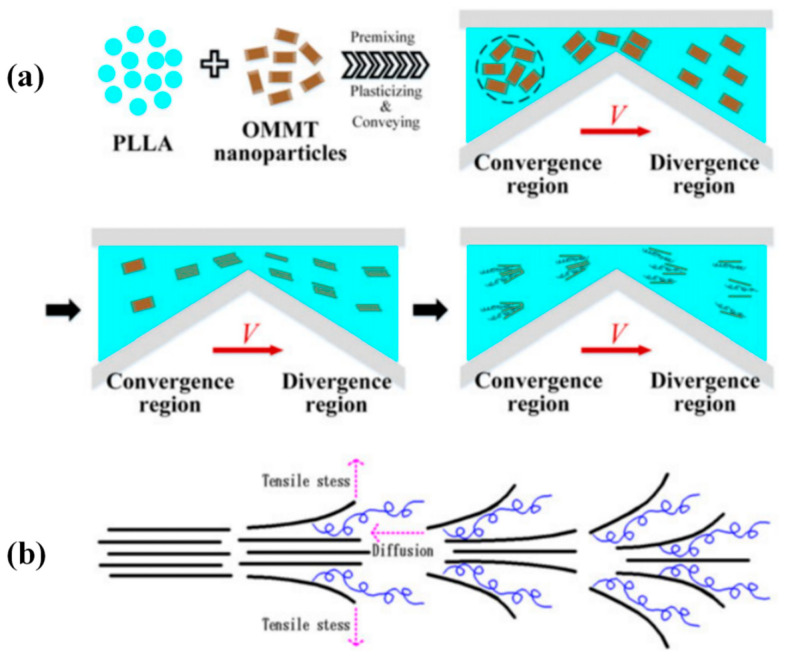
The Schematic illustration of (**a**) dispersion and exfoliation process of organically modified montmorillonite (OMMT) particles, and (**b**) double-side exfoliation mechanism. Reproduced with permission from [16]. Copyright 2017, John Wiley and Sons.

Wu et al. [53] prepared the PP/PS/MMT blends using ERE. With the addition of MMT nanofillers, the size of the PS dispersive phase was reduced, and compatibility of PP/PS improved. The synergy of extensional deformation and interfacial interaction enabled the MMT nanoparticles to exfoliate during processing. They further studied the in-situ exfoliation phenomenon in the poly-L-lactide (PLLA)/OMMT composite [16]. As demonstrated in Figure 10a, after premixing, the aggregated OMMT nanoparticles were first dispersed by repeated converging-diverging channels in ERE, then a double-side exfoliation of the OMMT nanoparticles took place under the perpendicular tensile stress and diffusion of PLLA chains, as shown in Figure 10b. This double-side exfoliation mechanism has also proven to be more effective than conventional layer-by-layer exfoliation mechanism in shear flow dominated processing devices in other polymer/OMMT composites. In ERE processed PLA/PBS/OMMT composites, Tan et al. [55] described the mechanism of the intercalation and exfoliation under elongation flow more intuitively with a simplified model, proving that this elongational flow induced dispersion and exfoliation mechanism could be applied in processing ternary polymer-inorganic composites. More research regarding the poly-L-lactide/poly ethylene glycol)/organomodified montmorillonite nanocomposites (PLLA/PEG/OMMT) were carried out by Wu et al. [15,56]. It was revealed that the increase in interlayer spacing of OMMT nanofiller under elongational flow significantly increased the nucleation density and nucleation rate in the composites, providing improved thermal stability. Zhang et al. [57] prepared the PP/OMMT composites using both ERE and TSE, and the comparative investigation showed that even at a high OMMT loading, nano-tactoids of clay were formed instead of intensive reaggregation proving that the double-sided exfoliation process induced by elongation flow is more effective.

It is widely recognized that nanofiller could accelerate the crystallization of polymers during processing, due to increased nucleating sites. Zhang et al. [58] studied PLA/TiO_2_ composites under elongational flow induced by VE. They found that instead of the rotation of aggregated particles in steady shear flow, the nanoparticles were separated and dispersed under repeated volume deformation through the converging channels in VE, leading to improved thermal stability, increased toughness and UV resistance. Jia et al. [59] compared the VE-extruded and SSE-extruded LDPE/nanoprecipitated calcium carbonate (NPCC) composites. VE-extruded samples presented better dispersion and distribution of nano-CaCO_3_ particles, leading to a 30% increase in elongation at break (from 810% to 840%) compared to SSE-extruded counterparts. Chen et al. [60] prepared a ternary blend of PLA/PBS and calcium sulfate whiskers (PLA/PBS/CSW) using VE. The well-distributed CSW improved the poor compatibility of PLA and PBS, and the finely dispersed and distributed PLA droplets in the PBS matrix induced by the elongational flow in VE resulted in improvements in the thermal resistance for PBS. 

Owing to the high aspect ratio and low compatibility with polymer blends, CNTs and multiwalled carbon nanotubes (MWCNTs) were easily aggregated during the processing in polymer blends, hindering the property improvement of such composites. Meng et al. [61] prepared the polypropylene/poly(ethylene-co-octene)/MWCNT composites (PP/POE/MWCNT) utilizing the ERE-induced elongational flow. The well-dispersed MWCNTs in the polymer matrix promoted significant improvements in heterogeneous nucleation, resulting in better tensile strength, flexibility and thermal stability of the ERE-extruded samples as compared to TSE-extruded samples.

Lin et al. [62] prepared the UHMWPE/OMMT composites using ERE and investigated the thermal-mechanical properties. In contrast to other polymer/OMMT composites, most of the nanoplates were well exfoliated and intercalated in the UHMWPE matrix under ERE-induced elongational flow, even at a high OMMT content. This might be due to the ultra-long chains and ultra-high entanglement density of UHMWPE, which increased the mean spacing distance, as simulated in previous theoretical work [27]. With a 1 wt% OMMT addition, the UHMWPE/OMMT composites exhibited an increase in impact strength from 127.3 kJ m^−2^ to 155.2 kJ m^−2^ as compared to neat UHMWPE.

As proven by the aforementioned studies in this section, the elongational flow dominated processing techniques could improve the dispersion and distribution of nanofillers as well as the crystallization and consequent performances of the targeted polymer/polymer blends in the polymer-inorganic composites.

### 3.3. Fiber-Reinforced Polymer Composites

Fiber-reinforced polymer composites possess many advantages such as light weight, recyclability and corrosion resistance [63,64]. However, the conventional shear flow dominated extrusion devices face some troubling issues, such as the attrition of fibers caused by the friction with the heated barrel, the poor orientation and distribution of fibers in the shear flow field, and limited fiber–polymer compatibility. In the search of solutions to these problems, researchers employed the elongational dominated flow in fiber-reinforced polymer composites processing.

**Figure 11:**
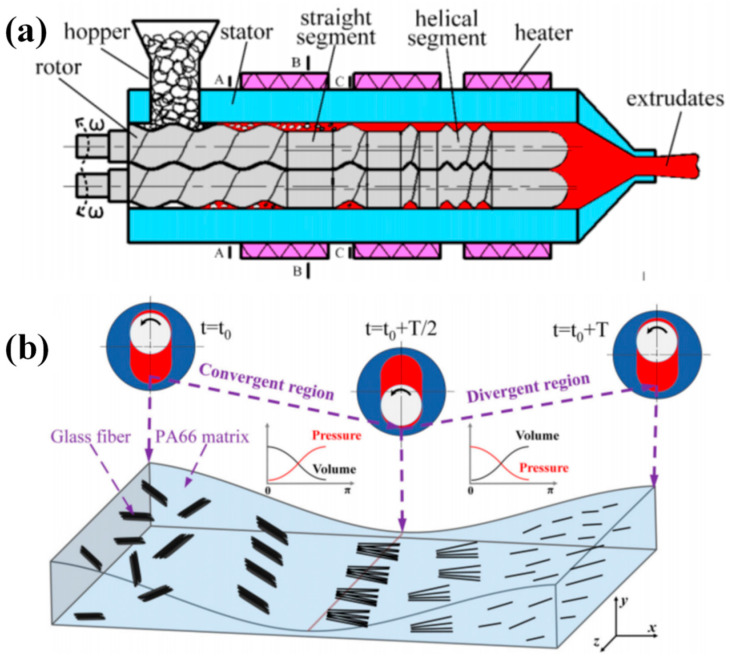
Schematic illustration of (**a**) overall structure of TERE. Reproduced under the CC BY-NC-ND license from [17]. and (**b**) the dispersion mechanism of glass fiber (GF) in the PA66 matrix. Reproduced with permission from [67]. Copyright 2020, John Wiley and Sons.

Wu et al. [65] fabricated the sisal fiber (SF)/PP composites under elongational flow field induced by VE. It was found that the simple addition of maleic anhydride-grafted polypropylene as a compatilizer would result in good performance of the SF/PP composites without the need for complex fiber treatment as in conventional extrusion methods [64,66]. Most recently, Wu et al. [17] and Guo et al. [67] investigated the glass fiber (GF) reinforced PA6 and PA66 composites using a novel TERE, as shown in Figure 11a. They found that the shear flow field formed in the conventional plastic molding devices such as SSE and TSE, can break up the agglomerates by applying excessive shearing intensity, but the subsequent low retention of the average fiber length, would lead to performance degradation. In contrast, under elongational flow field, induced by TERE, the fibers were well-dispersed and evenly distributed in polymer matrix, as demonstrated in Figure 11b. The average fiber lengths in TERE-extruded GF/PA6 and GF/PA66 composites were 2.7 and 3.2 times that of TSE-extruded samples. The increased fiber length retention resulted in substantial increase in impact strength, flexural strength, and tensile strength as well.

These studies demonstrated the distinct advantages of elongational flow dominated processing over conventional shear flow dominated techniques in providing good dispersion and length retention of fibers in fiber-reinforced polymer composites. It is expected to be a simple and feasible way to process expanded fiber-reinforced polymer composites such as carbon fibers, modification-free natural fiber composites and so on.

## 4. Conclusions and Prospects

For over half a century, researchers have realized the superiority of elongational flow field in polymer plasticizing and conveying process over shear flow field. Limited by the device design, the shear flow dominated plasticizing and conveying methods and related devices were still the dominant processing techniques for polymer and polymer composites. With the emerging theoretical studies and equipment designs in elongational flow dominated plasticizing and conveying technologies, as we summarized and discussed in this review, many of the critical issues in conventional screw-based extrusion devices such as long thermal-mechanical history, high energy consumption, and dependence on material properties have been solved with the application of EME replacements, vane extruders and eccentric rotor extruders. These methods and related devices also showed great potential to solve more specific problems like the dispersion and distribution of the polymer components, reaggregation of nanofiller and retention of fiber length.

Despite the great advancements that have been made in this vibrant field, numerous issues of the currently reported methods and devices still remain to be solved. First, as in EME-modified screw-extruders, repeated insertion of the EME would lead to severe pressure loss, thus the elongational flow dominated sections can’t totally replace the screw-based sections. Second, as in the vane extruder, the stiffness of the vanes becomes the limiting factor in applications in extreme conditions, and dead ends in the flow channel, though much smaller than TSE, still need structural optimization. Third, as for the eccentric rotor extruder, with increasing eccentricity and rotation speed, the power consumption also increased. The search for the balance between power consumption, throughput of the products, and products’ overall performance is still to be resolved. Last, future developments in the visualization of the plasticizing/conveying process and in-situ characterization techniques would help us further understand the intrinsic mechanisms and offer more efficient optimization of the processes. We believe that with these issues solved and upcoming developments in this field, the elongational flow dominated processing technologies will contribute more to the sustainable development of polymer processing industries.

## Data Availability

The data presented in this study are available on request from the corresponding author.
